# An Empirical Study on Corporate ESG Behavior and Employee Satisfaction: A Moderating Mediation Model

**DOI:** 10.3390/bs14040274

**Published:** 2024-03-26

**Authors:** Tianxing Zhang, Jun Zhang, Siyuan Tu

**Affiliations:** 1 Law School, Fudan University, Shanghai 200438, China; 2 Judicial Research Institute, Shanghai University of Political Science and Law, Shanghai 201701, China; zhangj@shupl.edu.cn; 3 Intellectual Property Research Institute, University of Science and Technology of China, Hefei 230026, China

**Keywords:** ESG behavior, employee satisfaction, executive environmental awareness, employee education, corporate governance

## Abstract

As the role of human capital in enhancing corporate value becomes increasingly prominent in the new economic era, employee satisfaction has garnered widespread attention in organizational behavior theory and business practices. However, constrained by the traditional governance model of “shareholder primacy”, which tends to view employees instrumentally, adverse effects on employee satisfaction and organizational identification persist. Currently, corporate ESG behaviors are flourishing in China, bringing profound and extensive transformations to economic and social sustainability. Yet, the research on whether and how corporate ESG behaviors improve employee satisfaction remains unclear. This study, based on data from the “China’s 100 Best Employers Award” and employing regression analysis on panel data from listed companies on the Shanghai and Shenzhen stock exchanges, reveals that corporate ESG behaviors have the potential to enhance employee satisfaction. Transparency in corporate environmental information and internal control mechanisms emerge as the primary means through which corporate ESG behaviors elevate employee satisfaction. Furthermore, heightened environmental awareness among executives and higher educational qualifications among employees strengthen the relationship between corporate ESG behaviors and employee satisfaction.

## 1. Introduction

In the new economic era, with human capital increasingly becoming a strategic resource for companies to gain sustained competitive advantage and with corporate governance objectives evolving from “shareholder primacy” to “stakeholderism”, the status of employees as core members of the enterprise is receiving heightened attention. Employees not only play a pivotal role in the day-to-day operations of the company but are also regarded as creators and beneficiaries of corporate culture and interests. However, at present, employee satisfaction in China is not as satisfactory as desired. According to statistical data, Chinese employee satisfaction is significantly lower than the global average. The primary reasons for this phenomenon include unmet expectations regarding interesting and well-paying jobs, as well as opportunities for career advancement [1]. Simultaneously, the widespread presence of gender bias against female employees and the lack of procedural justice exacerbate this situation [2]. Employee satisfaction not only directly correlates with an individual’s sense of personal fulfillment and happiness but also influences organizational behavior, job performance, and overall productivity. Low employee satisfaction not only significantly hampers employee performance, leading to an increase in the intention to leave [3], but also serves as a crucial factor contributing to employees’ mental health issues [4]. In response to this, the Chinese government has introduced a series of policy documents aimed at safeguarding employee salaries and welfare, with the hope of using policy measures to guide companies to prioritize employee well-being, effectively enhancing employee satisfaction. Companies are also actively supporting employee participation in management, supervision, and decision making [5], broadening channels for employees to express their opinions [6], positively impacting their job satisfaction. In recent years, research on employee satisfaction has primarily revolved around the relationship between employee satisfaction and job performance and company performance [7,8]. It has been found that higher job satisfaction plays a constructive role in improving both employee and organizational performance.

Against the backdrop of the increasing prominence of corporate social responsibility (CSR) and climate change issues, the ESG concept has garnered significant attention. ESG is a composite concept consisting of environmental, social, and governance elements, widely applied in areas such as financial investment, information disclosure, and corporate governance. ESG serves as both an evaluation criterion for measuring corporate sustainable development performance [9] and a practical method guiding the integration of sustainability into corporate investment decisions [10]. Specifically, “E” emphasizes business practices related to resource utilization and climate change mitigation, “S” focuses on interactions with stakeholders and social impacts, and “G” emphasizes sound governance structures and management practices aligned with the company’s best interests [11]. Since its formal proposal by the United Nations in 2004, ESG has evolved into a core component of modern corporate strategy [12]. Chinese companies, in response to the government’s “dual carbon goals” and pressures from market investors, have also adopted a series of ESG behaviors. Existing research primarily focuses on the influencing factors and consequences of corporate ESG behavior. The previous literature has extensively explored the determinants of corporate ESG performance from internal perspectives such as ownership structure and internal governance features [13,14]. In addition, executive team characteristics [15], the market environment of the country of operation, legal systems, and culture are also crucial factors influencing corporate ESG performance [16]. Existing research consistently confirms the positive impact of corporate ESG behavior on company value and financial performance growth. Superior ESG performance has been shown to reduce various market risks and capital costs, including legal risks [17], credit risks [18], overturning, to a certain extent, the initial assertions of a negative correlation between ESG performance and stock returns [19].

This study, combining panel data from listed companies on the Shanghai and Shenzhen stock exchanges in China with data from “China’s 100 Best Employers Award”, utilizes regression analysis to confirm that corporate ESG behavior significantly enhances employee satisfaction. Stability tests are employed to establish the reliability of the results. The results of mechanism analysis indicate that internal control mechanisms and transparency of external environmental information are the primary mediating factors through which corporate ESG behavior enhances employee satisfaction. Heterogeneity analysis further demonstrates that heightened environmental awareness among executives and higher employee educational qualifications strengthen the relationship between corporate ESG behavior and employee satisfaction. The findings of this research can provide policy insights for government bodies, industry organizations, and other market entities involved in promoting ESG development to enhance employee satisfaction in China.

This study makes several important theoretical contributions. Firstly, despite the rapid development of ESG-related research and significant progress, the understanding of the relationship between corporate ESG behaviors and employee attitudes remains unclear. There is limited recognition in the literature that employee satisfaction can be influenced by the organization’s implementation of ESG behaviors. Previous research has extensively examined the link between CSR and employee satisfaction [20,21]. While both emphasize the positive externalities brought to society by non-financial contributions [22], ESG and CSR exhibit essential distinctions. Unlike CSR, ESG entails a more comprehensive evaluation of a company’s sustainable performance [23], encompassing not only its social impact but also quantified analyses of environmental effects and transparency and efficiency in corporate governance. Therefore, the potential mechanisms underlying the “ESG behavior-employee satisfaction” relationship are more intricate. This study, by substituting ESG for CSR as a research variable, holds theoretical and practical significance. It is imperative to further explore the academic discourse on the relationship between ESG and employee satisfaction in the foundation of CSR, aligning with the evolving concepts of organizational sustainable governance to augment the marginal contribution of ESG research. Secondly, existing research indicates that organizations generally use intermediary mechanisms such as organizational justice [24] and organizational commitment [25] to positively influence employee satisfaction through CSR. However, the literature has not provided explanations or examinations of the potential mechanisms underlying the “ESG behavior-employee satisfaction” relationship. In contrast to CSR, which primarily shapes a positive brand image [26], ESG focuses on internal corporate governance. A robust internal control system can effectively integrate ESG issues, creating value for the company [27], and employees can participate in ESG decision making as governance entities. Corporate governance thus plays a crucial intermediary role between corporate ESG behavior and employee attitudes. This study identifies and introduces corporate governance as a potential intermediary factor, offering a more comprehensive exploration of internal control mechanisms and the external information environment, thereby elucidating how ESG behavior influences employee satisfaction at the organizational level and filling a theoretical gap. Thirdly, in analyzing the moderating factors of the relationship between CSR and employee satisfaction, existing research often emphasizes external objective factors such as procedural justice [28], compensation, and benefits [29]. Since CSR behaviors are typically seen as passive responses to external pressures like policy requirements and market forces, this study adopts a cognitive theory perspective, considering that corporate ESG behavior is a product of the interaction between organizational self-systems and the external environment influenced by cognitive processes. It underscores the crucial role of managerial environmental awareness in organizational decision making and execution. Fourthly, corporate human capital includes both senior management and ordinary employees, collectively influencing the realization and effectiveness of corporate ESG strategies. However, the existing literature predominantly focuses on studying the impact of executive team characteristics on environmental performance and social responsibility fulfillment [30]. There is limited research on the role of employees in the impact of corporate ESG behavior on employee satisfaction and their individual characteristics [31], with analysis primarily carried out from the perspective of employee perceptions of the company’s CSR activities [32]. This study enriches the research on the moderating factors in the impact of corporate ESG behavior on employee satisfaction by considering the educational level of employees. It breaks through the theoretical limitations often analyzed from the executive level, providing valuable insights for companies seeking to enhance employee satisfaction through ESG behavior.

Of course, this study faces certain potential limitations. Firstly, due to time and budget constraints, we used the “Top 100 Best Employers in China” as a measure of employee satisfaction. While this list is one of the most influential and authoritative awards in the field of Chinese employer satisfaction, reflecting the real situation of employee satisfaction in China, the research results may be biased due to the limitations of voluntary company participation and the coverage of listed companies. Secondly, for companies with different ownership structures and in different regions, differences in policy and market environments make the effects of corporate ESG behavior on employee satisfaction heterogeneous. Thirdly, there may be other factors influencing the impact of corporate ESG behavior on employee job satisfaction; however, this study only considers corporate governance behavior.

## 2. Theoretical Background and Hypotheses

### 2.1. Corporate ESG Behavior and Employee Satisfaction

In the era of the knowledge economy, the increasing importance of human capital in creating corporate value has drawn substantial attention from both academia and industry. The issue of employee satisfaction is a topic of widespread concern. Employee satisfaction, or “job satisfaction”, is typically regarded as the most crucial and frequently studied work attitude in the field of organizational behavior. Employee satisfaction refers to a positive or pleasant emotional state experienced by employees due to their assessment of their work or work experiences [33]. This sense of satisfaction, derived from work, encompasses both physiological and psychological aspects [34]. It not only reflects the extent to which employees’ actual experiences align with their expectations regarding the organization [35] but also correlates negatively with employees’ intentions to leave [36]. Employees who are dissatisfied with their work are more likely to exhibit a stronger inclination towards leaving their positions [37]. Conversely, when individuals are committed and loyal to the organization and the organization provides more opportunities for personal development, the higher the level of commitment between the organization and the employee, the lower the employee’s intention to leave [38]. Currently, discussions surrounding employee satisfaction focus on the factors influencing it and its outcomes. On the one hand, existing research explores factors influencing individuals’ attitudes toward work, such as the nature of the job, compensation, promotion opportunities, and relationships with colleagues. Among these, the most classic Two Factor Theory categorizes factors influencing employee motivation into motivator factors and hygiene factors. It posits that only motivator factors related to the nature of the job and intrinsic rewards can have a lasting impact on employees. When motivator factors are present, employees are more likely to feel satisfied and motivated intrinsically, thereby enhancing job performance. The absence of hygiene factors, which are related to working conditions and the external environment, may cause employee dissatisfaction, but their presence does not necessarily increase employee satisfaction and work motivation [39]. Existing empirical research broadly supports the notion that positive employee sentiments and high job satisfaction play a constructive role in enhancing corporate performance. Internally, employee-friendly organizations are closely associated with higher labor productivity efficiency [40]. These organizations incentivize innovation through the establishment of competitive welfare benefits [41], leading to improved organizational performance and business outcomes [42]. Moreover, differences in job satisfaction can impact a company’s investment efficiency, as high job satisfaction effectively mitigates moral hazards and adverse selection problems arising from information asymmetry, thereby enhancing investment efficiency [43]. Externally, higher employee satisfaction positively contributes to the long-term stock returns and shareholder returns of a company [44]. It can enhance the company’s external financing conditions and reduce debt financing costs [45].

ESG, as a new corporate development concept covering the core elements of the environment, the social element, and governance, promotes a shift in corporate objectives from maximizing value towards considering both economic and social/environmental values. Current research focuses on examining the degree of correlation between corporate ESG performance and financial performance. Most research conclusions indicate that positive ESG performance contributes positively to improving financial performance and corporate value [46]. Firstly, in terms of corporate financing activities, proactive ESG behavior helps to convey positive signals to the market regarding the company’s commitment to sustainable development [47], gaining recognition and financial support from investors and a wide range of stakeholders, alleviating corporate financing constraints [48]. Secondly, strong ESG performance contributes to shaping a robust internal control environment to effectively address operational, information, and compliance risks in daily business processes [49], as well as transformation risks faced during the sustainable development process, thereby enhancing the success rate of transformations [50]. Thirdly, ESG performance has a positive impact on corporate operational activities in terms of enhancing innovation levels and production efficiency [51]. Corporate ESG behavior can improve the company’s relationships with stakeholders, helping the company access diverse external information, knowledge, and financial support to promote technological innovation activities [52], thereby effectively increasing the company’s total factor productivity [53]. Finally, at the corporate investment level, ESG represents an investment philosophy that considers non-financial performance, such as environmental, social, and governance factors, along with traditional financial performance. Corporate ESG behavior can mitigate agency problems, reduce managerial opportunistic behavior, and minimize inefficient investments [54].

In the current context of sustainable transformation in economic and social development, as more and more companies implement ESG practices, the potential impact on employee satisfaction is growing. From a social and environmental perspective, CSR initiatives can translate into positive outcomes for employees [55]. The relationship between corporate fulfillment of environmental and social responsibilities and employee satisfaction can be explained, firstly, using the theory of “Social Identity”. Social identity refers to the self-image content that individuals derive from the social categories to which they perceive themselves as belonging [56]. When individuals recognize their membership in a specific social group and understand the emotions and values associated with being a group member [57], they tend to choose and endorse activities consistent with their social identity in organizational behavior [58]. This social identity is correlated with employees’ positive evaluations and identification with the organization [59]. As organizational identification grows, employees are more likely to endorse the organization’s values and practices, leading to increased loyalty and satisfaction. Therefore, employees who support ESG attitudes are more likely to recognize and endorse corporate ESG behavior, fostering positive social identity with the organization. This positive sentiment not only aids employees in actively supporting organizational norms and value goals but also contributes to their sense of satisfaction. Secondly, the theory of “Job Embeddedness” provides a theoretical perspective for understanding the relationship between corporate ESG behavior and employee satisfaction. Job embeddedness refers to the sum of various social, psychological, and economic factors that constrain employees within their current organization and job [60]. According to embeddedness theory, when employees have multiple connections with people in the organization and community, perceive a good fit with the organization and community, and realize that leaving would result in significant losses, their attachment and sense of belonging to the organization strengthen [61]. When organizations provide employees with opportunities to participate in ESG activities, employees not only feel satisfied with the chance to contribute to the community but also form close connections and higher compatibility with the organization, community, and surrounding environment [62]. This platform aligns organizational values with employees’ personal values, enhancing job compatibility and satisfaction [63]. Lastly, research indicates that stakeholders, including employees, are increasingly attentive to a company’s sustainable practices in the environmental and social domains [64]. Engaging in ESG activities not only enhances a company’s reputation but the noble mission of the company also inspires employees, fostering a stronger sense of pride among organizational members, promoting higher organizational identification [65]. Consequently, employees feel satisfied with their employment relationship.

In the realm of corporate governance, ESG behavior not only focuses on compliance but also involves embedding ESG principles into the existing organizational governance structure. On one hand, corporate ESG behavior in corporate governance primarily manifests as improvements in governance structure diversification and transparency. The implementation of ESG corporate governance principles and policies is expected to effectively reduce instances of employee discrimination [66], providing fair compensation, creating favorable working conditions, and fostering positive relationships among colleagues, demonstrating a commitment to ensuring employee welfare and thereby enhancing employee satisfaction. On the other hand, actively promoting employee stock ownership and involvement in corporate governance endows employees with dual roles as workers and owners. The establishment of “psychological ownership” contributes to strengthening employees’ organizational identification [67]. Leveraging the governance effects of employee stock ownership plans enhances employees’ willingness and ability for internal supervision, preventing executives from compromising employee welfare for personal gain or shareholder interests. In necessary instances, employees can exercise shareholder rights, for example, through voting, to curb such actions [68]. Therefore, we propose the following hypothesis:

**H1.**  *There is a positive correlation between corporate ESG behavior and employee satisfaction.*

### 2.2. The Mediating Role of Corporate Governance

Corporate governance mechanisms are typically categorized into internal control mechanisms and external information environments, with improvements in internal control quality and external information environments serving as crucial facets of ESG behavior in the corporate governance realm. Internal control systems refer to processes established by the board of directors, management, and all employees to provide reasonable assurance for achieving control objectives [69]. Implementing ESG behaviors can create a favorable external environment for the operation of internal control systems [70]. Integrating key performance indicators related to the environment, society, and governance into internal management systems can further enhance the effectiveness of ESG behaviors [71,72], ultimately leading to increased employee satisfaction. On the one hand, internal control serves as a robust mechanism for supervising and constraining management, preventing opportunistic behaviors such as adverse selection and moral hazards [73]. This ensures that management places greater emphasis on the interests of stakeholders, including employees [74]. On the other hand, effective internal control systems can streamline decision-making processes and corporate governance mechanisms, alleviating conflicts of interest among shareholders, management, and employees caused by uncoordinated governance structures. This enables employees’ opinions to be promptly heard and acknowledged by management. Moreover, a sound internal control mechanism can provide employees with a positive workplace culture and environment, opportunities for professional and personal growth, and fair and welfare-oriented treatment [75].

In addition to internal control mechanisms, the external information environment also provides a pathway for enhancing employee satisfaction through corporate ESG behavior. Corporate adherence to ESG behaviors can offer additional non-financial information, improving the efficiency of stock market information and mitigating information asymmetry [76]. In practice, ESG information disclosure by companies typically follows ESG disclosure frameworks and the standards set by international organizations or third-party institutions. Research indicates that this third-party supervisory mechanism, focusing on processes rather than content, facilitates more effective information disclosure by companies [77]. A more transparent disclosure of ESG information enables institutional investors to engage in governance and encourages companies to take more proactive ESG actions. Currently, investors in the capital market increasingly consider a company’s ESG performance as a factor in their investment decisions, which are driven not only by individual investor values [78] but also by the desire to avoid litigation risks from stakeholders [79] and potential damage to interests due to environmental risks [80]. However, low-quality information disclosure makes it challenging for external investors to monitor management’s self-interested decision making [81]. The improvement of the information environment is conducive to institutional investors obtaining a comprehensive view of a company’s sustainable development capabilities from more abundant and transparent ESG information. Institutional investors can leverage their relative advantages in governance cost–benefit analysis, shareholding proportions, information collection, and investment research analysis capabilities [82]. They act as governance entities with supervisory roles [83], exerting pressure on companies by exercising shareholder rights, actively fulfilling ESG responsibilities, and ultimately enhancing employee satisfaction. Based on this, we propose the following hypothesis:

**H2.**  *Internal control mechanisms and the external information environment play a mediating role in the relationship between ESG behavior and employee satisfaction. ESG behavior enhances employee satisfaction by improving internal control efficiency and enhancing the external information environment.*

### 2.3. The Modulating Role of Top Management’s Environmental Awareness

Traditionally, outstanding managerial capabilities have been a crucial criterion for evaluating the performance of corporate executives. However, with ESG gradually replacing shareholder returns as the global consensus for assessing corporate governance standards, the significance of top management’s ESG expertise and green awareness in influencing employee satisfaction and sustainable corporate development has become increasingly paramount. As a vital concept in the field of psychology, awareness refers to an individual’s capacity for perceiving, judging, reasoning, and constructing ideas in response to environmental or organizational stimuli. It forms the foundation that supports individuals in decision-making and behavioral implementation. As an integral component of top management’s ESG awareness, environmental awareness not only mirrors differences in executives’ recognition of the importance of environmental issues in management [84] but also significantly reflects their attitudes toward economic, social, and environmental sustainability. The formation of top management’s environmental awareness is influenced by various external factors. The economic value of environmental measures constitutes a primary factor motivating managers to actively respond to environmental issues [85]. Legitimacy pressures [86], personal beliefs, and moral and ethical principles also impact their adoption of environmentally friendly practices in business activities [87].

The subjective cognition of corporate executives directly influences the formulation and implementation of corporate ESG strategic actions. A fundamental prerequisite for effectively managing ESG issues at the business level is a fundamental shift in business mindset [88]. Firstly, managerial decision making is a process based on selective perception and is influenced by personal values [89]. Thus, employee satisfaction is highly dependent on the ethical sense and concern of management. Executives with a pro-social inclination typically implement welfare policies favorable for employees [90]. Secondly, according to the Attention-Based View (ABV), attention is a crucial resource within organizations, and the time and energy that organizational managers allocate to specific issues are extremely limited [91]. Cognitive structures further restrict and influence what issues and solutions they can focus on [92]. Under bounded rationality, the strategic decision-making and behavioral choices of a company often depend on where managers choose to focus their attention. Therefore, executives’ awareness of environmental concepts and their interpretation of environmental policies can significantly impact the implementation of corporate ESG behaviors. Generally speaking, for companies to enhance employee satisfaction through the implementation of ESG behaviors, they must overcome the limitations of managerial attention and focus on improving employee welfare and compensation. Stakeholder studies confirm this idea; only stakeholder groups that receive managerial attention are likely to be considered in business decision making [93]. Thirdly, based on the “Cognitive-Emotional Processing System” framework [94], situational stimuli formed by individual subjective perceptions, including thoughts, cognition, and planning, can stimulate individuals’ “cognitive-emotional units” [95]. Therefore, positive environmental orientation cognition possessed by top executives can influence individuals’ processing and coping behaviors towards environmental information, promoting the formation of pro-environmental behaviors among employees. When employees perceive that the company is making efforts to enhance environmental welfare, it increases their organizational identification and, consequently, their sense of satisfaction. Therefore, we propose the following hypothesis:

**H3.**  *The environmental awareness of top executives in listed companies moderates the relationship between corporate ESG behavior and employee satisfaction. A higher level of executive environmental awareness strengthens the promotion relationship between corporate ESG behavior and employee satisfaction.*

### 2.4. The Moderating Role of Employee Educational Attainment

As a crucial component of corporate human capital [96], employees’ educational backgrounds constitute the informal institutional framework of a company, providing a soft constraint on corporate ESG behaviors. Currently, the interaction between corporate human capital and corporate performance has become a focal point of research. The qualifications of employees exert a significant impact on economic factors such as financial performance, operational strategies, employee job satisfaction, and innovation activities within a company [97], contributing to the enhancement of core competitiveness and value creation. Additionally, employee qualifications are integral to a company’s ESG performance and fulfillment of social responsibilities, thereby improving the effectiveness of the implementation of corporate ESG policies.

The education received by employees plays a formative role in shaping their cognitive frameworks and value systems [98]. Generally, employees with higher educational qualifications are more likely to be influenced by concepts such as “social responsibility” and “employee rights” during the learning process, thus exhibiting higher expectations in terms of values and self-realization [99]. Primarily, the quality education received by employees establishes a soft constraint on their behavioral norms, prompting a conscious resistance against organizational actions that deviate from their moral standards. When highly educated employees ascend to management positions and contribute to the formulation of company strategies, they demonstrate a heightened ability to comprehend and consider the demands of various stakeholders in a rational and objective manner [100]. This facilitates a better balance between operational efficiency and employee welfare, ultimately enhancing employee satisfaction. Secondly, the ESG-related knowledge and performance capabilities possessed by highly educated employees form a solid foundation for corporations to fulfill their ESG responsibilities. Higher levels of education equip employees with an increased understanding of ESG information pertaining to legal regulations, ethical considerations, and more, enabling corporations to navigate ESG risks and challenges effectively and to address the demands of stakeholders, including employees [101]. Furthermore, highly educated employees generally exhibit a stronger organizational identification with corporate ESG behaviors. The quality of employees influences their organizational commitment and moral alignment [102]. Studies indicate that individuals with higher levels of education place greater emphasis on CSR issues [103] and develop a more nuanced perception of CSR [104]. Differences in the education levels of employees significantly impact the perception of distributive justice and procedural justice [105]. Therefore, highly educated employees tend to identify more with organizational ESG behaviors, thereby promoting an enhancement in their own satisfaction. Based on these considerations, we posit the following hypothesis:

**H4.**  *The educational qualifications of employees moderate the relationship between corporate ESG behaviors and employee satisfaction, with higher educational qualifications strengthening the positive impact of corporate ESG behaviors on employee satisfaction.*

## 3. Research Design

### 3.1. Sample Selection and Data Sources

This study utilizes panel data constructed from A-share listed companies on the Shanghai and Shenzhen stock exchanges for the period spanning 2015 to 2022, facilitating regression analysis. One major obstacle in empirically testing the hypotheses presented above is the lack of authoritative standards for measuring employee satisfaction in China. To address this issue, we observed that in previous research, the “The 100 Best Companies to Work For” jointly released by the Great Place to Work Institute and Fortune magazine has been widely used as an alternative indicator for measuring employee satisfaction [106,107,108]. However, since this list does not disclose companies from mainland China, in this study, we constructed a measure of employee satisfaction using data obtained from two sources. Firstly, data were sourced from the “China’s 100 Best Employers Award” published by Zhaopin Limited from 2015 to 2022. This list annually selects the top 100 employers in mainland China and is jointly conducted by Zhaopin Limited, a leading internet recruitment and job-seeking company in China, in collaboration with Peking University’s Institute of Social Science Survey and its National Development Institute, China Labor Economics Society, and other professional institutions. The Social Research Center of Peking University serves as a co-initiator and, together with other professional institutions, forms the selection committee, establishing application regulations, selection rules, and evaluation indicators; convening expert supervision of the evaluation process; analyzing data; and compiling reports. Drawing on Western research achievements, the evaluation system was constructed, which integrates with global mainstream practices and possesses Chinese characteristics. Specifically, the evaluation system comprises four dimensions and six subdivided sub-dimensions, including organizational structure, growth system, incentive system, and cultural system (see Table 1). The “China’s 100 Best Employers Award” employs a diverse evaluation system where the final score is composed of employee surveys, anonymous ratings by an enterprise human resources review panel, evaluations by an expert review panel, and qualification reviews. All relevant information is publicly released on the website and is subject to public scrutiny, ensuring the objectivity, scientific basis, and reliability of the evaluation results. Additionally, this list has received support from the Harvard Business Review in China and has been widely disseminated by numerous mainstream media, further enhancing its prestige. Currently, the list has become one of the most influential and authoritative awards in the field of Chinese employer satisfaction, drawing significant attention from shareholders, management, employees, and the media. Therefore, it is reasonable to assume that the “China’s 100 Best Employers Award” provides an authoritative, neutral, and quantifiable measure of employee satisfaction. Notably, compared to other measures of job satisfaction, this list has several advantages. Firstly, it offers a comprehensive assessment of employee satisfaction. A company’s score in the “China’s 100 Best Employers Award” is derived not only from online surveys of company employees but also from ratings by thousands of anonymous enterprise human resources managers, expert scholars, non-governmental organizations, and media professionals on the evaluation panel. Therefore, it considers not only observable practices of the company but also conducts in-depth grassroots analysis based on extensive employee surveys. For instance, KLD heavily relies on self-disclosure by companies and uses a narrative-based scoring method, making its scores susceptible to manipulation by companies and potentially failing to reflect employees’ true sentiments [109]. Directly asking respondents about their job satisfaction through survey questionnaires is also a crucial way to measure employee satisfaction. Meanwhile, relying solely on questionnaires may face criticisms of treating satisfaction as a single construct and having low reliability [110]. Secondly, the selection committee of the “Top 100 Best Employers in China” has been officially publishing the annual list on its website since 2011, providing a longer sample interval that helps to ensure that result accuracy is not influenced by specific periods or market conditions [111]. Thirdly, since 2015, the number of companies participating in the selection has continuously increased, with 101,143 companies applying for selection in 2023, where on average, over 50% of the companies were publicly listed in previous years [112]. This comprehensive coverage of listed companies minimizes the self-selection bias resulting from companies nominating themselves, with the enlarged sample size alleviating potential biases. Secondly, employee satisfaction data are collected through a keyword search of CSR reports. ESG behavior data are sourced from the Sino-Securities Index ESG Ratings. Financial and governance data at the corporate level are extracted from the China Stock Market & Accounting Research Database (CSMAR database). Simultaneously, the sample of listed companies during the study period undergoes the following treatments: exclusion of financial industry companies, removal of data with missing financial information, and exclusion of data from companies labeled as ST (Special Treatment) or *ST (Special Treatment for two consecutive years). Additionally, this study employs winsorization on continuous variables within the sample.

### 3.2. Model Specification

To examine the impact of ESG behavior on employee satisfaction, it is imperative to construct a model. Diverging from conventional models, both dependent variables in this study, namely employee satisfaction, are binary variables ranging from 0 to 1. As such, it is necessary to employ a logistic regression model. The following foundational regression model has been established in this study:

(1) $$P(New\_col_{it}=1)=\alpha_{0}+\alpha_{1}ESG_{it}+\alpha_{k}\sum Control_{it}+\gamma_{t}+\mu_{i}+\varepsilon_{it}$$

(2) $$P(Disclose2_{it}=1)=\alpha_{0}+\alpha_{1}ESG_{it}+\alpha_{k}\sum Control_{it}+\gamma_{t}+\mu_{i}+\varepsilon_{it}$$

Here, the subscript *i* denotes the firm, and *t* represents time. ESG stands for the independent variable of ESG behavior. “new_col” and “disclose2” are the dependent variables representing employee satisfaction in this study. Control encompasses a series of control variables, $\gamma_{t}$ denotes time effects, $\mu_{i}$ represents industry effects, and $\varepsilon_{it}$ accounts for the random disturbance term. Within this context, $\alpha_{1}$ signifies the core estimated parameters, and $\gamma_{t}$ represents the direct effect of ESG behavior on employee satisfaction. According to the research hypothesis, a significant and positive relationship is anticipated; specifically, ESG behavior is expected to have a significant positive impact on employee satisfaction.

In order to further investigate the mechanisms through which ESG behavior operates, building upon the baseline regression and drawing inspiration from the mediation models proposed by Wang and Ge (2022) and Wang et al., (2023), two mediating pathways related to corporate information transparency and internal control are examined [113,114]. The constructed mediation model is as follows:

(3) $$Med_{it}=\beta_{0}+\beta_{1}ESG_{it}+\beta_{m}\sum Control_{it}+\gamma_{t}+\mu_{i}+\varepsilon_{it}$$

(4) $$P(New\_col_{it}=1)=\varphi_{0}+\varphi_{1}Med_{it}+\varphi_{m}\sum Control_{it}+\gamma_{t}+\mu_{i}+\varepsilon_{it}$$

(5) $$P(Disclose2_{it}=1)=\omega_{0}+\omega_{1}Med_{it}+\omega_{m}\sum Control_{it}+\gamma_{t}+\mu_{i}+\varepsilon_{it}$$

Here, $Med_{it}$ represents the mediating variable, which includes corporate information transparency (trans) and internal control (lndb). To ensure clarity in the examination results, the testing of mediating mechanisms is conducted separately. $\beta_{0}$ and $\varphi_{0}$ are constants, $\beta_{1}$ represents the coefficient for ESG behavior, and $\varphi_{1}$ and $\omega_{1}$ are the coefficients for the mediating variables. The interpretation of the remaining variables is consistent with Equation (1). Prior to interpreting the results of the mediating mechanisms, the focus lies on the significance of $\beta_{1}$. If this coefficient is significant, it indicates that the mediating mechanisms of ESG behavior are functioning as expected.

### 3.3. Variable Definitions

#### 3.3.1. Dependent Variables

Employee satisfaction. Following the approach of Liu and Lin (2020), our criteria for measuring employee satisfaction consist of two indicators. Firstly, this involves confirming whether a company made it to the list of “China’s 100 Best Employers Award” from 2015 to 2022. If the company is included in the list of “China’s 100 Best Employers Award”, the value is assigned as 1; otherwise, it is designated as 0. Secondly, the assessment involves collecting information on whether the company disclosed information on employee rights protection and safety production in the respective year. This information serves as an indicator of employee satisfaction within the company [115].

#### 3.3.2. Independent Variable

ESG behavior. Employing the method of Zhang et al., (2023), ESG behavior is measured using the Sino-Securities Index ESG Ratings. Within the Sino-Securities Index ESG Ratings, the environmental (E) dimension includes environmental management systems, green business objectives, and green products. The social (S) dimension encompasses institutional systems, health and safety, and social contributions. The corporate governance (G) dimension involves institutional development, governance structure, and operational activities [116]. Specifically, ESG ratings (C to AAA, nine-grade ratings) are assigned values from 1 to 9, reflecting the company’s ESG performance and measuring ESG behavior.

#### 3.3.3. Mediating Variables

Corporate information transparency (trans). Drawing on the studies by Bushman et al., (2004) and Xin et al., (2014), an indicator evaluation system is constructed, including earnings quality (DD), disclosure score (DSCORE), analyst coverage (ANALYST), analyst earnings forecast accuracy (ACCURACY), and auditor perspective (BIG4) [117,118]. The average percentile rank of these five variables is used to measure corporate information transparency. If any of these transparency variables are missing for a listed company, trans is set to be equal to the average percentile rank of the remaining variables. A higher trans value indicates greater corporate transparency.

Regarding the measurement of internal control (lndb), following the approach of Li and Shi (2019), the “DIB China Listed Companies Internal Control Index” is utilized to measure the degree of internal control governance. This index is represented by the natural logarithm of the internal control index plus 1 [119].

#### 3.3.4. Control Variables

Following the practices of Barasa et al., (2017), Rong et al., (2017), and Zuo and Lin (2022), financial and corporate governance data are employed as control variables [120,121,122]. Specifically, control variables include equity concentration, profitability, financial leverage, corporate growth, cash holdings, company size, age at establishment, dual roles of executives, board size, and proportion of independent directors. The main variable definitions in this paper are shown in Table 2.

## 4. Empirical Analysis

### 4.1. Descriptive Statistical Analysis

Based on the descriptive statistics presented in Table 3, the core variables in this study—ESG, new_col, and disclose2—exhibit significant differences in their maximum and minimum values, indicating substantial variations in ESG behavior and employee satisfaction across different companies. The descriptive results of the control variables fall within the expected ranges. Moreover, for over half of the control variables, the mean values exceed the standard deviations, suggesting relatively small coefficients of variation and high data stability.

### 4.2. Basic Regression Analysis

According to the regression results presented in Table 4, columns (1) and (2) report the estimates of the Logit model, incorporating industry and time effects. Unlike OLS estimation, Logit models do not report goodness-of-fit in software. Reviewing the regression results from these two columns, the regression coefficients for ESG are both significantly positive, signifying a positive influence on employee satisfaction. Specifically, in the new_col model, the regression coefficient for ESG is 0.111, which is statistically significant at the 1% level based on significance testing. This indicates that for every 1% improvement in corporate ESG governance, employee satisfaction increases by 0.111%. There is a significant positive correlation between the two. Additionally, the 95% confidence interval for the regression coefficient of 0.111 in column (1) is [0.0735–0.1490]. Moreover, in column (2), we chose a different employee satisfaction metric and found that for every 1% improvement in corporate ESG governance, employee satisfaction increases by 0.064%, maintaining a stable positive impact. The 95% confidence interval for the regression coefficient of 0.0642 in column (2) is [0.0577–0.0706]. The regression results above robustly indicate that ESG behavior effectively enhances employee satisfaction. The regression results for control variables are also consistent with theoretical expectations, with the majority of control variables being significantly positive, suggesting a positive impact on employee satisfaction within the broader operational context of the companies.

### 4.3. Robustness Checks

#### 4.3.1. Alternative Independent Variables

To conduct robustness checks, this study adopts a method of replacing independent variables. If the replacement of independent variables maintains the consistency of the regression coefficients and their significance, the robustness of the baseline regression is affirmed. Specifically, drawing inspiration from the approach of Lei et al., (2023), Bloomberg ESG is used as an alternative to the Sino-Securities Index ESG Ratings [123]. As seen in the regression results in Table 4, columns 1 and 2, the regression coefficients for Dcg are 0.045 and 0.143, respectively, with both being significant at the 1% level. This affirms the robustness of the baseline regression, indicating that ESG behavior significantly promotes employee satisfaction.

#### 4.3.2. Alternative Dependent Variables

To test robustness, this study adopts a method of replacing dependent variables. If the replacement of dependent variables maintains the consistency of the regression coefficients and their significance, the robustness of the baseline regression is affirmed. In the baseline regression, the variable was set to 1 if the company’s CSR report disclosed either “employee rights” or “safety production”, and it was 0 otherwise. For this robustness check, a new dummy variable, disclose1, is set if the variable meets the criteria of either employee rights or safety production disclosure. As shown in the regression results in Table 4, column 3, the regression coefficient for ESG is 0.0665, being significant at the 1% level. This supports the robustness of the baseline regression conclusions.

#### 4.3.3. Addressing Temporal Lag Issues

Given the potential time lag in employees’ responses to corporate ESG behavior, this study considers the issue of temporal lag. After the implementation of ESG behaviors by companies, employees may not necessarily respond in the same period, introducing a certain degree of temporality and uncertainty into the employee satisfaction metric used in this study. Therefore, the study advances the employee satisfaction variable used in the baseline regression by one period using data from year t+1 for the analysis. As seen in the regression results in Table 5, columns 4 and 5, the regression coefficients for ESG exhibit consistent direction and significance, suggesting the robustness of the findings from the baseline regression.

### 4.4. Mechanism Analysis

#### 4.4.1. Corporate Information Transparency Mechanism

Columns (1) to (3) in Table 5 examine the mediating role of corporate information transparency in the impact of ESG behavior. In column 1, the regression coefficient for ESG is 0.0051, being significant at the 1% level, indicating that ESG behavior significantly promotes corporate information transparency. The coefficients for “trans” in columns 2 and 3 are significantly positive, suggesting that an improvement in corporate information transparency contributes to increased employee satisfaction. These results validate the mediating role of corporate information transparency in the relationship between ESG behavior and employee satisfaction.

#### 4.4.2. Corporate Internal Control Mechanism

Columns (4) to (6) in Table 6 test the mediating role of corporate internal control in the impact of ESG behavior. In column 4, the regression coefficient for ESG is 0.0058, being significant at the 1% level, indicating that ESG behavior significantly promotes corporate internal control. The coefficients for “lndb” in columns 5 and 6 are significantly positive, suggesting that an enhancement in corporate internal control contributes to increased employee satisfaction. These results indicate the mediating role of corporate internal control in the relationship between ESG behavior and employee satisfaction.

## 5. Heterogeneity Analysis

### 5.1. Heterogeneous Impact of Environmental Awareness among Corporate Executives

To examine whether the environmental awareness of corporate executives moderates the relationship between ESG behavior and employee satisfaction, this study follows the approach of Duriau et al., (2007) and Xi Longsheng and Zhao Hui (2022). It constructs keywords reflecting the degree of environmental concern by the management, such as energy conservation, environmental strategy, environmental philosophy, environmental education, environmental training, environmental technology development, environmental audit, and environmental facilities. The frequency of these keywords in the annual reports is used to measure the environmental awareness of corporate executives, and the data are log-transformed for analysis [124,125]. The environmental awareness data of executives are grouped heterogeneously, dividing the companies into two groups based on high and low executive environmental awareness and estimating the relationship separately. As seen in the results in the first and second columns of Table 7, the regression coefficient for ESG is significantly positive in the high executive environmental awareness group, indicating that ESG behavior significantly enhances employee satisfaction. This result suggests that in companies where executive environmental awareness is high, the implementation of ESG behavior is more likely to lead to employee satisfaction. This effect is not as pronounced in the low executive environmental awareness group. Therefore, it is possible to conclude that the environmental awareness of corporate executives plays a crucial role in moderating the relationship between ESG behavior and employee satisfaction.

### 5.2. Heterogeneous Impact of Employee Education on ESG Behavior and Employee Satisfaction

To examine whether differences in employee education levels can positively moderate the relationship between ESG behavior and employee satisfaction, we use employee education data for heterogeneity analysis. Based on the composition of employee education, we categorize employees into three groups: no education, diploma or bachelor’s degree, and graduate education. To highlight the presence of highly educated employees, we consider employees with graduate education as those with high education levels. We calculate the ratio of employees with graduate education to the total workforce for each company, group these ratios based on the annual industry median, and create two subgroups: high employee education and low employee education. This grouping method helps eliminate the size effect of the overall education level of employees. The higher the ratio, the more highly educated employees a company has, indicating a higher proportion of highly educated employees. This is illustrated in the first and second columns of Table 8. In the heterogeneous subgroup analysis of companies with highly educated employees, the regression coefficient for ESG is significantly positive. Conversely, in the subgroup of companies with lower educated employees, the coefficient for ESG is not significant. This suggests that employee education enhances the positive impact of ESG behavior on employee satisfaction. In conclusion, the higher the education level of employees, the more evident the promoting effect of ESG behavior on employee satisfaction. This observation may be attributed to the theoretical understanding that higher educated employees are more aware of ecological conservation. They tend to identify with and appreciate environmental practices such as ESG behavior, thus strengthening the moderating effect of ESG behavior on employee satisfaction.

## 6. Conclusions and Recommendations

### 6.1. Conclusions

This study conducted empirical analysis on the impact of corporate ESG behavior on employee satisfaction using data from 2015 to 2022 for listed companies on the Shanghai and Shenzhen stock exchanges. Additionally, the study constructed a moderating and mediating effect model to analyze the mediating roles of internal control effectiveness and external information transparency in this relationship, as well as the moderating roles of environmental awareness among top executives and employee education levels. The results indicate that (1) corporate ESG behavior significantly enhances employee satisfaction; (2) corporate environmental information transparency and internal control mechanisms play mediating roles in the relationship between corporate ESG behavior and employee satisfaction; and (3) higher levels of environmental awareness among top executives and employee education strengthen the positive impact of corporate ESG behavior on employee satisfaction. Our research findings are robust and may provide valuable insights for corporate decision-makers.

The marginal contributions of this paper are as follows: Firstly, the existing literature on corporate ESG behavior primarily focuses on the economic consequences such as impacts on company value, financial performance, and financing costs. This paper addresses the inadequacy of studies concerning the relationship between corporate ESG behavior and employee work attitudes. It provides explanations and validation for the potential mechanisms of the “ESG behavior-employee satisfaction” relationship. Secondly, this study takes a perspective rooted in cognitive theory, asserting that corporate ESG behavior is a product of the interaction between organizational self-system and the external environment under the influence of cognitive processes. It emphasizes the crucial role of environmental awareness and cognition in organizational decision making and execution. Thirdly, this research enriches the study of moderating factors in the impact of corporate ESG behavior on employee satisfaction from the perspective of employees’ educational levels. It breaks through the theoretical limitations of the existing literature that mainly focuses on executive team characteristics, providing valuable insights for companies aiming to enhance employee satisfaction through ESG behavior.

### 6.2. Recommendations

Based on the above research conclusions, the following policy recommendations are proposed:

(1) Encourage and support companies to practice ESG behavior. The implementation of ESG behavior not only helps companies establish a positive image and gain sustainable market competitiveness but also plays a crucial role in enhancing employee organizational identity and satisfaction. While ESG behavior primarily relies on voluntary actions by companies and investors, the government has played a key driving force in CSR in China. The government should establish clear guidance frameworks for corporate ESG actions through legislation and regulations. Additionally, various policy tools such as tax incentives, fiscal subsidies, and credit support should be utilized to encourage and guide companies to actively implement ESG behavior. Furthermore, intermediary institutions are a critical part of the ESG ecosystem, involving ESG information disclosure, an objective evaluation system, and the integration of market data. Intermediary institutions should provide rating and evaluation services for ESG companies, compiling ESG indices and offering data integration services to support investors’ decision making, to enhance the credibility of information disclosure, and to help investors to identify and mitigate ESG investment risks. Institutional investors can promote companies’ attention and improvement of their ESG performance through their investment decisions and exercise of shareholder rights.
(2) Strengthen the new mechanism of corporate ESG behavior promoting employee satisfaction. Under the theoretical framework of ESG promoting employee satisfaction, fully leverage the advantages of corporate ESG behavior in strengthening internal control effectiveness and promoting market information effects. Regarding internal control, integrate ESG assessment content into internal control systems, identify and evaluate potential ESG risks in the company’s production and operation processes, conduct regular ESG management supervision through internal control audits, and ensure that ESG goals are implemented through the company’s internal management system. Simultaneously, strengthen internal ESG management and training during the internal control audit process, increase executive awareness of ESG, and promote the implementation of ESG-related policies and management processes. Concerning external information transparency, companies should enhance supervision and control over the quality of ESG disclosure information; comply with ESG disclosure rules, policies, and related advocacy principles; and ensure the accuracy, timeliness, and completeness of disclosure information.
(3) Incorporate ESG principles into human resources development strategies. Establish a human resources management concept and system guided by ESG, improve executive and employee training systems, and enhance the ESG leadership of boards of directors and executives. To improve the ESG awareness of the management team, companies can choose to externally recruit talents with expertise and experience in ESG to join the board of directors. Alternatively, they can shape and influence the careers of managers to help them to understand the strategic significance of sustainable development and to cultivate managers with sustainable development thinking.

## Figures and Tables

**Table 1:** Evaluation system.

Dimensions	Sub-Dimensions	Evaluation Index
Organizational system	Work environment	(1) Sense of pride (2) Management identification (3) Feeling of support (4) Sense of security (5) Job burnout
	Organizational management	(1) Sense of duty (2) Communication (3) Management system identity (4) Sense of importance (5) Perfection of talent system (6) Perception of promotion equity (7) Sense of respect (8) Challenging (9) Incentivization (10) Value equivalence
Growth system	Growth and development	(1) Sense of achievement (2) Sense of importance (3) Challenging (4) Career confusion (5) Opportunity concerns
Incentive system	Salary and welfare	(1) Welfare satisfaction (2) Pay system popularization (3) Value equivalence (4) Performance rationality (5) Salary rationality (6) Competitiveness
Cultural system	Employer culture	(1) Value recognition (2) Sense of importance (3) Sense of purpose (4) Loyalty (5) Feeling of warmth (6) Sense of respect (7) Sense of worth (8) Cultural experience (9) Workplace enjoyment (10) Sense of participation
	Employer image	(1) Empathy (2) Sense of responsibility (3) Sense of pride (4) Product identity (5) Recommendation level (6) Sense of trust (7) Sense of belonging

Data So. Data resource: 2023 China’s 100 Best Employers Award.

**Table 2:** Definitions and explanations of variables.

Variables	Variable Symbol	Variable Definition	Data Source
Employee satisfaction	new_col	If a company is included in the annual list of the top 100 employers, the value is set to 1; otherwise, it is set to 0	Manually organizing the content of the “China’s 100 Best Employers Award” to obtain information
	disclose2	If a company’s CSR report discloses items related to “employee rights” and “safety production”, the value is set to 1; otherwise, it is set to 0	Manually organizing the content of the “CSR Report” to obtain information
ESG behavior	ESG	Sino-Securities Index ESG Ratings	Acquisition from Sino-Securities Index Information Service (Shanghai) Co., Ltd. (Shanghai, China) disclosure
Information environment transparency	Trans	The information transparency index evaluation system is constructed, and the value is computed accordingly	Acquisition from CSMAR database and WIND database
Internal controls	Lndb	The natural logarithm of the DIB internal control and risk management database	Acquisition from DIB disclosrue
Size of company	Size	The natural logarithm of the total assets of the firm	Acquisition from CSMAR database
Financial leverage	Lev	Ratio of total liabilities to total assets	
Profitability	Roa	Return on total assets	
Cash-holding level	Cash	The ratio of cash flows from operating activities to total assets	
Growth of enterprises	Growth	Adopts the natural logarithm of the number of board members	
Board size	Board	Adopts the natural logarithm of the number of board members	
Ratio of independent directors	Indep	The ratio of the number of independent directors to the total number of board members	
Dual posts in one	Dual	The indicator takes a value of 1 when the roles of chairman and CEO are held by the same individual, being 0 otherwise	
Shareholding of the largest shareholder	Top1	The ownership ratio of the largest shareholder in the company	
Age of establishment	Age	The natural logarithm of the number of years since the company’s establishment	

**Table 3:** Descriptive statistics of key variables.

Variable Symbol	Observed Value	Mean Value	Standard Deviation	Minimum Value	Maximum Value
new_col	20,590	0.0110733	0.1046482	0	1
disclose2	20,590	0.5694512	0.4951651	0	1
ESG	20,590	73.50565	5.110355	58.63	84.49
size	20,590	22.35712	1.288317	19.7446	26.4523
lev	20,590	0.4094915	0.1898036	0.051455	0.901682
roa	20,590	0.0423827	0.0651994	−0.373035	0.247308
cash	20,590	0.0522081	0.0644566	−0.172921	0.26687
growth	20,590	0.1584652	0.3739178	−0.657557	4.02421
board	20,590	2.108195	0.1955394	1.60944	2.70805
indep	20,590	37.80894	5.370982	28.57	60
dual	20,590	0.3056338	0.4606865	0	1
top1	20,590	33.26022	14.51543	8.0204	74.8237
age	20,590	2.976887	0.2865628	2.07944	3.61092

**Table 4:** Results of basic regression analysis.

	(1)	(2)
	new_col	disclose2
ESG	0.111 ***	0.0642 ***
	(0.0192)	(0.00329)
size	1.225 ***	0.351 ***
	(0.0812)	(0.0173)
lev	2.033 ***	−0.0400
	(0.675)	(0.109)
roa	1.284	−0.718 **
	(1.905)	(0.294)
cash	2.953 **	1.095 ***
	(1.453)	(0.267)
growth	−0.115	−0.168 ***
	(0.221)	(0.0436)
board	−0.597	0.142
	(0.419)	(0.105)
indep	−0.0224	−0.00719 **
	(0.0147)	(0.00361)
dual	0.237	−0.00229
	(0.186)	(0.0337)
top1	0.000378	−0.000714
	(0.00539)	(0.00112)
age	1.282 ***	0.130 **
	(0.342)	(0.0579)
Constant	−57.63	−13.06 ***
	(809.8)	(0.493)
Time effects	Control	Control
Industry effects	Control	Control
N	16,316	20,590

Note: **, *** indicate significance at the 5%, and 1% levels, respectively. Standard errors are reported in parentheses, and the same convention applies throughout.

**Table 5:** Robustness check results.

	(1)	(2)	(3)	(4)	(5)
	new_col	disclose2	disclose1	F.new_col	F.disclose2
Dcg	0.0458 ***	0.143 ***			
	(0.0117)	(0.00744)			
ESG			0.0665 ***	0.134 ***	0.0635 ***
			(0.00408)	(0.0213)	(0.00349)
size	0.998 ***	0.0525	0.312 ***	1.219 ***	0.366 ***
	(0.103)	(0.0388)	(0.0226)	(0.0887)	(0.0195)
lev	2.238 ***	−0.577 **	0.271 **	2.049 ***	−0.158
	(0.767)	(0.238)	(0.136)	(0.745)	(0.122)
roa	1.924	−1.209 *	−0.563	2.502	−0.827 **
	(2.113)	(0.670)	(0.367)	(2.153)	(0.328)
cash	4.331 ***	−0.372	0.996 ***	2.356	0.733 **
	(1.625)	(0.568)	(0.332)	(1.603)	(0.299)
growth	−0.116	−0.165 **	−0.187 ***	0.00713	−0.146 ***
	(0.222)	(0.0774)	(0.0496)	(0.231)	(0.0467)
board	−0.506	−0.127	−0.100	−0.382	0.123
	(0.460)	(0.195)	(0.135)	(0.447)	(0.117)
indep	0.000945	−0.0121 *	−0.0156 ***	−0.0122	−0.00673 *
	(0.0162)	(0.00668)	(0.00464)	(0.0155)	(0.00404)
dual	0.117	−0.142 *	−0.0329	0.146	−0.0310
	(0.216)	(0.0753)	(0.0426)	(0.204)	(0.0375)
top1	−0.00130	−0.00152	−0.00472 ***	−0.00234	0.00108
	(0.00610)	(0.00222)	(0.00142)	(0.00591)	(0.00124)
age	1.194 ***	0.249 **	0.220 ***	1.416 ***	0.00258
	(0.375)	(0.122)	(0.0727)	(0.382)	(0.0639)
Constant	−45.48	−3.740 ***	−10.39 ***	−60.32	−12.75 ***
	(581.1)	(0.960)	(0.632)	(835.3)	(0.547)
Time effects	Control	Control	Control	Control	Control
Industry effects	Control	Control	Control	Control	Control
N	4663	6232	20,572	13,443	16,938

Note: *, **, *** indicate significance at the 10%, 5%, and 1% levels, respectively. Standard errors are reported in parentheses, and the same convention applies throughout.

**Table 6:** Mediation mechanism test results.

	(1)	(2)	(3)	(4)	(5)	(6)
	trans	new_col	disclose2	lndb	new_col	disclose2
ESG	0.0051 ***			0.00583 ***		
	(0.0002)			(0.000228)		
trans		0.929 **	0.405 ***			
		(0.408)	(0.0898)			
lndb					1.161 *	0.291 ***
					(0.641)	(0.0975)
size	0.0777 ***	1.302 ***	0.382 ***	0.00837 ***	1.343 ***	0.412 ***
	(0.0012)	(0.0840)	(0.0184)	(0.00117)	(0.0800)	(0.0170)
lev	−0.073 ***	1.684 ***	−0.332 ***	0.0160 **	1.493 **	−0.325 ***
	(0.0086)	(0.641)	(0.109)	(0.00773)	(0.641)	(0.108)
roa	0.920 ***	1.477	−0.184	0.691 ***	1.262	−0.0203
	(0.0233)	(1.876)	(0.303)	(0.0210)	(1.912)	(0.300)
cash	0.153 ***	3.103 **	0.946 ***	−0.0919 ***	2.847 **	1.016 ***
	(0.0212)	(1.450)	(0.268)	(0.0191)	(1.442)	(0.268)
growth	0.0161 ***	−0.213	−0.212 ***	0.0447 ***	−0.252	−0.219 ***
	(0.00342)	(0.210)	(0.0433)	(0.00308)	(0.215)	(0.0435)
board	0.00104	−0.419	0.202 *	0.0103	−0.350	0.196 *
	(0.00810)	(0.408)	(0.105)	(0.00729)	(0.410)	(0.105)
indep	−0.000268	−0.00760	−0.00188	0.000102	−0.00590	−0.00149
	(0.000279)	(0.0142)	(0.00360)	(0.000251)	(0.0143)	(0.00359)
dual	0.00644 **	0.124	−0.0104	0.00301	0.137	−0.00444
	(0.00268)	(0.185)	(0.0338)	(0.00241)	(0.186)	(0.0337)
top1	2.80 × 10^−5^	0.000870	0.00113	0.000541 ***	−0.000211	0.000684
	(8.78 × 10^−5^)	(0.00536)	(0.00112)	(7.89 × 10^−5^)	(0.00541)	(0.00112)
age	−0.038 ***	1.348 ***	0.128 **	0.00263	1.369 ***	0.137 **
	(0.00463)	(0.330)	(0.0586)	(0.00415)	(0.335)	(0.0582)
Constant	−1.653 ***	−52.56	−9.684 ***	5.755 ***	−60.84	−12.11 ***
	(0.0364)	(876.7)	(0.484)	(0.0328)	(777.5)	(0.754)
Time effects	Control	Control	Control	Control	Control	Control
Industry effects	Control	Control	Control	Control	Control	Control
N	20,238	16,048	20,233	20,288	16,087	20,284

Note: *, **, *** indicate significance at the 10%, 5%, and 1% levels, respectively. Standard errors are reported in parentheses, and the same convention applies throughout.

**Table 7:** Heterogeneous results of executive environmental awareness.

	High Executive Environmental Awareness		Low Executive Environmental Awareness	
	new_col	disclose2	new_col	disclose2
	(1)	(2)	(3)	(4)
ESG	0.00101 ***	0.0128 ***	0.000264	0.00124
	(0.000203)	(0.000985)	(0.000261)	(0.00117)
size	0.0171 ***	0.0737 ***	0.00202	0.0185
	(0.00102)	(0.00496)	(0.00324)	(0.0146)
lev	−0.00870	−0.00146	0.0165	0.0320
	(0.00681)	(0.0330)	(0.0122)	(0.0550)
roa	−0.0241	−0.0799	0.0340	0.00433
	(0.0183)	(0.0889)	(0.0209)	(0.0940)
cash	0.0386 **	0.247 ***	−0.00724	0.0266
	(0.0168)	(0.0814)	(0.0181)	(0.0817)
growth	0.00156	−0.0417 ***	−0.00308	−0.0246 **
	(0.00273)	(0.0132)	(0.00265)	(0.0119)
board	−0.00496	0.0153	−0.00223	0.0345
	(0.00650)	(0.0315)	(0.0122)	(0.0552)
indep	0.000360	−0.00198 *	−0.000725 **	0.00206
	(0.000226)	(0.00109)	(0.000362)	(0.00163)
dual	0.00415 *	−0.00322	0.000822	0.00473
	(0.00213)	(0.0103)	(0.00330)	(0.0149)
top1	−3.97 × 10^−5^	−0.000289	7.56 × 10^−5^	−0.00146
	(6.98 × 10^−5^)	(0.000339)	(0.000203)	(0.000914)
age	0.00985 ***	0.0263	−0.0223	0.392 ***
	(0.00360)	(0.0174)	(0.0291)	(0.131)
Constant	−0.470 ***	−2.168 ***	0.0301	−1.239 **
	(0.0289)	(0.140)	(0.108)	(0.487)
Time effects	Control	Control	Control	Control
Industry effects	Control	Control	Control	Control
N	10,528	10,528	10,067	10,067

Note: *, **, *** indicate significance at the 10%, 5%, and 1% levels, respectively. Standard errors are reported in parentheses, and the same convention applies throughout. The same below.

**Table 8:** Heterogeneous results of employee education.

	High Employee Education		Low Employee Education	
	new_col	disclose2	new_col	disclose2
	(1)	(2)	(3)	(4)
ESG	0.00123 ***	0.0136 ***	0.000278	0.00109
	(0.000225)	(0.000882)	(0.000192)	(0.00126)
size	0.0210 ***	0.0727 ***	0.00448 *	0.0385 **
	(0.00113)	(0.00441)	(0.00253)	(0.0165)
lev	0.00394	−0.00736	−0.0112	0.000645
	(0.00754)	(0.0296)	(0.00956)	(0.0626)
roa	−0.000333	−0.133 *	−0.00120	0.0416
	(0.0203)	(0.0796)	(0.0158)	(0.104)
cash	0.0512 ***	0.276 ***	0.00662	−0.0272
	(0.0186)	(0.0730)	(0.0134)	(0.0878)
growth	−0.00242	−0.0483 ***	−0.00465 **	0.00837
	(0.00301)	(0.0118)	(0.00204)	(0.0133)
board	−0.00408	0.0256	−0.0215 **	−0.0205
	(0.00699)	(0.0274)	(0.00969)	(0.0634)
indep	−1.73 × 10^−5^	−0.00328 ***	−0.000398	−0.000182
	(0.000247)	(0.000968)	(0.000282)	(0.00185)
dual	0.00641 ***	−0.00224	−0.000822	−0.0169
	(0.00239)	(0.00937)	(0.00246)	(0.0161)
top1	−5.92 × 10^−5^	−0.000116	−0.000142	−0.00133
	(7.71 × 10^−5^)	(0.000302)	(0.000154)	(0.00101)
age	0.0174 ***	0.0205	−0.00143	0.230
	(0.00410)	(0.0161)	(0.0219)	(0.143)
Constant	−0.581 ***	−2.225 ***	−0.0388	−0.985 *
	(0.0323)	(0.127)	(0.0832)	(0.545)
Time effects	Control	Control	Control	Control
Industry effects	Control	Control	Control	Control
N	12,701	12,701	7894	7894

Note: *, **, *** indicate significance at the 10%, 5%, and 1% levels, respectively. Standard errors are reported in parentheses, and the same convention applies throughout. The same below.

## Data Availability

Data are contained within the article.

## References

[1] Zhang X., Kaiser M., Nie P., Sousa-Poza A. (2019). Why are Chinese workers so unhappy? A comparative cross-national analysis of job satisfaction, job expectations, and job attributes. PLoS ONE.

[2] Ngo H.Y., Foley S., Ji M.S., Loi R. (2014). Work satisfaction of Chinese employees: A social exchange and gender-based view. Soc. Indic. Res..

[3] Coomber B., Barriball K.L. (2007). Impact of job satisfaction components on intent to leave and turnover for hospital-based nurses: A review of the research literature. Int. J. Nurs. Stud..

[4] Nadinloyi K.B., Sadeghi H., Hajloo N. (2013). Relationship between job satisfaction and employees mental health. Procedia Soc. Behav. Sci..

[5] Zhu Y., Xie Y., Warner M., Guo Y. (2015). Employee participation and the influence on job satisfaction of the ‘new generation’ of Chinese employees. Int. J. Hum. Resour. Manag..

[6] Nawakitphaitoon K., Zhang W. (2021). The effect of direct and representative employee voice on job satisfaction in China: Evidence from employer-employee matched data. Int. J. Hum. Resour. Manag..

[7] Judge T.A., Thoresen C.J., Bono J.E., Patton G.K. (2001). The job satisfaction—Job performance relationship: A qualitative and quantitative review. Psychol. Bull..

[8] Bakotić D. (2016). Relationship between job satisfaction and organisational performance. Econ. Res. Ekonomska Istraživanja.

[9] Hřebíček J., Soukopová J., Trenz O. (2014). Current trends of economic modelling of sustainable corporate performance and reporting—Review and research agenda. Procedia Econ. Financ..

[10] Skroupa C.P. Define Metrics for ESG, CSR and the Like, and You’ll Grab Wall Street’s Attention. https://www.forbes.com/sites/christopherskroupa/2018/02/06/define-metrics-for-esg-csr-and-the-like-and-youll-grab-wall-streets-attention/?sh=4b8874105444.

[11] Huang D.Z. (2019). Environmental, social and governance (ESG) activity and firm performance: A review and consolidation. Acc. Financ..

[12] Tsang Y., Fan Y., Feng Z. (2023). Bridging the gap: Building environmental, social and governance capabilities in small and medium logistics companies. J. Environ. Manag..

[13] Abeysekera A.P., Fernando C.S. (2020). Corporate social responsibility versus corporate shareholder responsibility: A family firm perspective. J. Corp. Financ..

[14] Hsu P.-H., Liang H., Matos P. (2023). Leviathan Inc. and Corporate Environmental Engagement. Manag. Sci..

[15] Cronqvist H., Yu F. (2017). Shaped by their daughters: Executives, female socialization, and corporate social responsibility. J. Financ. Econ..

[16] Ferrell A., Liang H., Renneboog L. (2016). Socially responsible firms. J. Financ. Econ..

[17] Hong H., Liskovich I. Crime, Punishment and the Halo Effect of Corporate Social Responsibility. SSRN Paper 2015. https://www.nber.org/system/files/working_papers/w21215/w21215.pdf.

[18] Jiraporn P., Jiraporn N., Boeprasert A., Chang K. (2014). Does Corporate Social Responsibility (CSR) Improve Credit Ratings? Evidence from Geographic Identification. Financ. Manag..

[19] Hong H., Kacperczyk M. (2009). The price of sin: The effects of social norms on markets. J. Financ. Econ..

[20] Rahman S., Haski-Leventhal D., Pournader M. (2016). The effect of employee CSR attitudes on job satisfaction and organizational commitment: Evidence from the Bangladeshi banking industry. Soc. Responsib. J..

[21] Closon C., Leys C., Hellemans C. (2015). Perceptions of corporate social responsibility, organizational commitment and job satisfaction. Manag. Res. J. Iberoam. Acad. Manag..

[22] Chouaibi S., Chouaibi J. (2021). Social and ethical practices and firm value: The moderating effect of green innovation: Evidence from international ESG data. Int. J. Ethics Syst..

[23] Li T.T., Wang K., Sueyoshi T., Wang D.D. (2021). ESG: Research Progress and Future Prospects. Sustainability.

[24] Tziner A., Oren L., Bar Y., Kadosh G. (2011). Corporate social responsibility, organizational justice and job satisfaction: How do they interrelate, if at all?. Rev. Psicol. Trab. Organ..

[25] Asrar-ul-Haq M., Kuchinke K.P., Iqbal A. (2017). The relationship between corporate social responsibility, job satisfaction, and organizational commitment: Case of Pakistani higher education. J. Clean. Prod..

[26] Hsu K.T. (2012). The advertising effects of corporate social responsibility on corporate reputation and brand equity: Evidence from the life insurance industry in Taiwan. J. Bus. Ethics.

[27] Harasheh M., Provasi R. (2023). A need for assurance: Do internal control systems integrate environmental, social, and governance factors?. Corp. Soc. Responsib. Environ. Manag..

[28] Murshed F., Sen S., Savitskie K., Xu H. (2021). CSR and job satisfaction: Role of CSR importance to employee and procedural justice. J. Mark. Theory Pract..

[29] Chepkwony P.K., Kemboi A., Muange R.M. (2015). Moderating effect of csr orientation on the relationship between internal csr practices and employee job satisfaction. Glob. J. Hum. Resour. Manag..

[30] Vlachos P.A., Panagopoulos N.G., Rapp A.A. (2013). Feeling Good by Doing Good: Employee CSR-Induced Attributions, Job Satisfaction, and the Role of Charismatic Leadership. J. Bus. Ethics.

[31] Bauman C.W., Skitka L.J. (2012). Corporate social responsibility as a source of employee satisfaction. Res. Organ. Behav..

[32] Singhapakdi A., Lee D.-J., Sirgy M.J., Roh H., Senasu K., Yu G.B. (2019). Effects of perceived organizational CSR value and employee moral identity on job satisfaction: A study of business organizations in Thailand. Asian J. Bus. Ethic.

[33] Locke E.A., Dunnette M.D. (1976). The nature and causes of job satisfaction. Handbook of Industrial and Organizational Psychology.

[34] Hoppock R. (1935). Job Satisfaction.

[35] Collins W.C., Raju N.S., Edwards J.E. (2000). Assessing differential functioning in a satisfaction scale. J. Appl. Psychol..

[36] Yurchisin J., Park J., Brien M.O. (2010). Effects of ideal image congruence and organizational commitment on employee intention to leave. J. Retail. Consum. Serv..

[37] Cotton J.L., Tuttle J.M. (1986). Employee turnover: A meta-analysis and review with implications for research. Acad. Manag. Rev..

[38] Porter L.W., Steers R.M., Mowday R.T., Boulian P.V. (1974). Organizational commitment, job satisfaction, and turnover among psychiatric technicians. J. Appl. Psychol..

[39] Herzberg F., Bernard M., Narnara S. (1959). The Motivation to Work.

[40] Cao Z., Rees W. (2020). Do Employee-Friendly Firms Invest More Efficiently? Evidence from Labor Investment Efficiency. J. Corp. Financ..

[41] Chen C., Chen Y., Hsu P.H., Podolski E.J. (2016). Be nice to your innovators: Employee treatment and corporate innovation performance. J. Corp. Financ..

[42] Ahmad M.R., Raja R. (2021). Employee Job Satisfaction and Business Performance: The Mediating Role of Organizational Commitment. Vis. J. Bus. Perspect..

[43] Arvidsson S., Eierle B., Hartlieb S. Job Satisfaction and Investment Efficiency—Evidence from Crowdsourced Employer Reviews. https://www.sciencedirect.com/science/article/pii/S0263237322001463.

[44] Kessler S.R., Lucianetti L., Pindek S., Zhu Z., Spector P.E. (2020). Job Satisfaction and Firm Performance: Can Employees’ Job Satisfaction Change the Trajectory of a Firm’s Performance?. J. Appl. Soc. Psychol..

[45] Chi W., Chen Y. (2021). Employee satisfaction and the cost of corporate borrowing. Financ. Res. Lett..

[46] Friede G., Busch T., Bassen A. (2015). ESG and Financial Performance: Aggregated Evidence from More than 2000 Empirical Studies. J. Sustain. Financ. Invest..

[47] Ashwin Kumar N.C., Smith C., Badis L., Wang N., Ambrosy P., Tavares R. (2016). ESG factors and risk-adjusted performance: A new quantitative model. J. Sustain. Financ. Invest..

[48] Raimo N., Caragnano A., Zito M., Vitolla F., Mariani M. (2021). Extending the benefits of ESG disclosure: The effect on the cost of debt financing. Corp. Soc. Responsib. Environ. Manag..

[49] McAfee A., Brynjolfsson E. (2008). Investing in the IT That Makes a Competitive Difference. Harv. Bus. Rev..

[50] Zhou G., Liu L., Luo S. (2022). Sustainable development, ESG performance and company market value: Mediating effect of financial performance. Bus. Strat. Environ..

[51] Sun H., Bai T., Fan Y., Liu Z. (2024). Environmental, social, and governance performance and enterprise sustainable green innovation: Evidence from China. Corp. Soc. Responsib. Environ. Manag..

[52] Lin Y., Fu X., Fu X. (2021). Varieties in state capitalism and corporate innovation: Evidence from an emerging economy. J. Corp. Financ..

[53] Deng X., Li W., Ren X. (2023). More sustainable, more productive: Evidence from ESG ratings and total factor productivity among listed Chinese firms. Financ. Res. Lett..

[54] Samet M., Jarboui A. (2017). How does corporate social responsibility contribute to investment efficiency?. J. Multinatl. Financ. Manag..

[55] Chatzopoulou E.-C., Manolopoulos D., Agapitou V. (2021). Corporate Social Responsibility and Employee Outcomes: Interrelations of External and Internal Orientations with Job Satisfaction and Organizational Commitment. J. Bus. Ethics.

[56] Turner J.C., Oakes P.J. (1986). The significance of the social identity concept for social psychology with reference to individualism, interactionism and social influence. Br. J. Soc. Psychol..

[57] Tajfel H. (1972). Some developments in European social psychology. Eur. J. Soc. Psychol..

[58] Mael F., Ashforth B.E. (1992). Alumni and their alma mater: A partial test of the reformulated model of organizational identification. J. Organ. Behav..

[59] Ashforth B.E., Mael F. (1989). Social identity theory and the organization. Acad. Manag. Rev..

[60] Mitchell T.R., Holtom B.C., Lee T.W., Sablynski C.J., Erez M. (2001). Why people stay: Using job embeddedness to predict voluntary turnover. Acad. Manag. J..

[61] Chan W.L., Ho J.A., Sambasivan M., Ng S.I. (2019). Antecedents and outcome of job embeddedness: Evidence from four and five-star hotels. Int. J. Hosp. Manag..

[62] Ghosh D., Gurunathan L. (2014). Linking Perceived Corporate Social Responsibility and Intention to Quit: The Mediating Role of Job Embeddedness. Vis. J. Bus. Perspect..

[63] Villanova P., Bernardin H.J., Johnson D.L., Dahmus S.A. (1994). The validity of a measure of job compatibility in the prediction of job performance and turnover of motion picture theater personnel. Pers. Psychol..

[64] Meng Z., Zhou Y., Li E.Y., Peng X., Qiu R. (2023). Environmental and economic impacts of drone-assisted truck delivery under the carbon market price. J. Clean. Prod..

[65] Jones D.A. (2010). Does serving the community also serve the company? Using organizational identification and social exchange theories to understand employee responses to a volunteerism programme. J. Occup. Organ. Psychol..

[66] Grove H., Clouse M. (2018). Focusing on sustainability to strengthen corporate governance. Corp. Gov. Sustain. Rev..

[67] Pierce J.L., Rubenfeld S.A., Morgan S. (1991). Employee ownership: A conceptual model of process and effects. Acad. Manag. Rev..

[68] Chen H.L., Huang Y.S. (2006). Employee stock ownership and corporate RandD expenditures: Evidence from Taiwan’s information-technology industry. Asia Pac. J. Manag..

[69] COSO COSO Internal Control-Integrated Framework Principles. www.coso.org/Documents/COSO-ICIF-11x17-Cube-Graphic.pdf”www.coso.org/Documents/COSO-ICIF-11x17-Cube-Graphic.pdf.

[70] Moffitt J.S., Patin J.C.A., Watson L. (2023). Corporate Environmental, Social, and Governance (ESG) Performance and the Internal Control Environment. Account. Horiz..

[71] Gebhardt M., Thun T.W., Seefloth M., Zülch H. (2023). Managing Sustainability—Does the Integration of Environmental, Social and Governance Key Performance Indicators in the Internal Management Systems Contribute to Companies’ Environmental, Social and Governance Performance?. Bus. Strategy Environ..

[72] Wang P., Bu H., Liu F. (2022). Internal control and enterprise green innovation. Energies.

[73] Narayanan V.G. (1994). An analysis of auditor liability rules. J. Account. Res..

[74] Aggarwal R.K., Samwick A.A. (2006). Empire-builders and shirkers: Investment, firm performance, and managerial incentives. J. Corp. Financ..

[75] Chen J. (2007). A Study of Employee Engagement within a Chinese Context. Doctoral Dissertation.

[76] He F., Qin S., Liu Y., Wu J. (2022). CSR and idiosyncratic risk: Evidence from ESG information disclosure. Financ. Res. Lett..

[77] Darnall N., Ji H., Iwata K., Arimura T.H. (2022). Do ESG reporting guidelines and verifications enhance firms’ information disclosure?. Corp. Soc. Responsib. Environ. Manag..

[78] Amel-Zadeh A., Serafeim G. (2018). Why and How Investors Use ESG Information: Evidence from a Global Survey. Financ. Anal. J..

[79] Cerqueti R., Ciciretti R., Dalò A., Nicolosi M. (2021). ESG Investing: A Chance to Reduce Systematic Risk. J. Financ. Stab..

[80] Fernando C.S., Sharfman M.P., Uysal V.B. (2017). Corporate Environmental Policy and Shareholder Value: Following the Smart Money. J. Financ. Quant. Anal..

[81] Biddle G.C., Hilary G., Verdi R.S. (2009). How does financial reporting quality relate to investment efficiency?. J. Account. Econ..

[82] De La Cruz A., Medina A., Tang Y., Owners of the World’s Listed Companies OECD Capital Market Series. https://papers.ssrn.com/sol3/papers.cfm?abstract_id=3475351f.

[83] Black B.S. (1991). Agents watching agents: The promise of institutional investor voice. UCLA Law Rev..

[84] Analoui F., Karami A. (2002). How chief executives’ perception of the environment impacts on company performance. J. Manag. Dev..

[85] Roberts S., Lawson R., Nicholls J. (2006). Generating Regional-Scale Improvements in SME Corporate Responsibility Performance: Lessons from Responsibility Northwest. J. Bus. Ethics.

[86] Bansal P., Roth K. (2000). Why companies go green: A model of ecological responsiveness. Acad. Manag. J..

[87] Ajzen I., Fishbein M. (1980). Understanding Attitudes and Predicting Social Behavior.

[88] Sharma S., Pablo A.L., Vredenburg H. (1999). Corporate environmental responsiveness strategies: The importance of issue interpretation and organisational context. J. Appl. Behav. Sci..

[89] Hambrick D.C., Mason P.A. (1991). Upper echelons: The organization as a reflection of its top managers. Acad. Manag. Rev..

[90] Feng M., Ge W., Ling Z., Loh W.T. (2023). Prosocial CEOs, corporate policies, and firm value. Rev. Account. Stud..

[91] Jeremy G. (2018). Do Boards of Directors Influence Corporate Sustainable Development? An Attention-Based Analysis. Bus. Strategy Environ..

[92] Ocasio W. (1997). Towards an attention-based view of the firm. Strateg. Manag. J..

[93] Gadenne D.L., Kennedy J., McKeiver C. (2009). An Empirical Study of Environmental Awareness and Practices in SMEs. J. Bus. Ethics.

[94] Mischel W., Shoda Y. (1995). A cognitive-affective system theory of personality: Reconceptualizing situations, dispositions, dynamics, and invariance in personality structure. Psychol. Rev..

[95] Mischel W. (1973). Toward a cognitive social learning reconceptualization of personality. Psychol. Rev..

[96] Schultz T.W. (1960). Capital Formation by Education. J. Political Econ..

[97] Dyer W.G., Denison D.R. (1991). Corporate Culture and Organizational Effectiveness. Adm. Sci. Q..

[98] Luthar H.K., Dibattista R.A., Gautschi T. (1997). Perception of What the Ethical Climate is and What it Should be: The Role of Gender, Academic Status, and Ethical Education. J. Bus. Ethics..

[99] Elias R.Z. (2004). An Examination of Business Students’ Perception of Corporate Social Responsibilities before and after Bankruptcies. J. Bus. Ethics.

[100] Ghardallou W. (2022). Corporate Sustainability and Firm Performance: The Moderating Role of CEO Education and Tenure. Sustainability.

[101] Tsang Y., Fan Y., Feng Z., Li Y. (2024). Examining supply chain vulnerability via an analysis of ESG-prioritized firms amid the Russian-Ukrainian conflict. J. Clean. Prod..

[102] Hidalgo-Peñate A., Nieves J., Padrón-Robaina V. (2022). The influence of employees’ knowledge, organisational commitment, and culture on the innovativeness of vocational educational. Knowl. Manag. Res. Pract..

[103] Rosati F., Costa R., Calabrese A., Pedersen E.R.G. (2018). Employee attitudes towards corporate social responsibility: A study on gender, age and educational level differences. Corp. Soc. Responsib. Environ. Manag..

[104] Quazi A.M. (2003). Identifying the determinants of corporate managers’ perceived social obligations. Manag. Decis..

[105] Tessema M.T., Tsegai G., Ready K., Embaye A., Windrow B. (2014). Effect of employee background on perceived organizational justice: Managerial implications. Int. Rev. Adm. Sci..

[106] Faleye O., Trahan E.A. (2011). Labor-friendly corporate practices: Is what is good for employees good for shareholders?. J. Bus. Ethics.

[107] Bae K., Kang J., Wang J. (2011). Employee Treatment and Firm Leverage: A Test of the Stakeholder Theory of Capital Structure. J. Financ. Econ..

[108] Edmans A., Li L., Zhang C. (2014). Employee Satisfaction, Labor Market Flexibility, and Stock Returns around the World.

[109] Edmans A. (2011). Does the stock market fully value intangibles? Employee satisfaction and equity prices. J. Financ. Econ..

[110] Oshagbemi T. (2000). Gender differences in the job satisfaction of university teachers. Women Manag. Rev..

[111] Edmans A. (2012). The link between job satisfaction and firm value, with implications for corporate social responsibility. Acad. Manag. Perspect..

[112] Xu H., Ni X., Li C., Liu Y. (2020). Job satisfaction and firm leverage: Evidence from the “China’s best employer award 100” winners. China J. Account. Res..

[113] Wang F., Ge X. (2022). Can Low-carbon Transition Impact Employment—Empirical Evidence from Low-carbon City Pilot Policy. China Ind. Econ..

[114] Wang Y., Wei S., He X., Gu H. (2023). Environmental regulation and entrepreneurial activity: Evidence from the low-carbon city pilot policy in China. Sustain. Cities Soc..

[115] Liu J., Lin S. (2020). Employee Satisfaction and Firms’ Technological Innovation. J. Ind. Technol. Econ..

[116] Zhang C., Li L., Boxin L. (2023). The impact of ESG performance on the financing efficiency of enterprises in strategic emerging industries. J. Financ. Account..

[117] Bushman R.M., Piotroski J.D., Smith A.J. (2004). What determines corporate transparency?. J. Account. Res..

[118] Qingquan X., Dongmin K., Ying H. (2014). Corporate transparency and stock price volatility. Financ. Res..

[119] Yingmei L., Qi S. (2019). Can internal control promote the innovation performance of enterprises?. Sci. Res. Manag..

[120] Barasa L., Knoben J., Vermeulen P., Kimuyu P., Kinyanjui B. (2017). Institutions, resources and innovation in East Africa: A firm level approach. Res. Policy.

[121] Rong Z., Wu X., Boeing P. (2017). The Effect of Institutional Ownership on Firm Innovation: Evidence from Chinese Listed Firms. Res. Policy.

[122] Zuo Z., Lin Z. (2022). Government R&D subsidies and firm innovation performance: The moderating role of accounting information quality. J. Innov. Knowl..

[123] Lei N., Miao Q., Yao X. (2023). Does the implementation of green credit policy improve the ESG performance of enterprises? Evidence from a quasi-natural experiment in China. Econ. Model..

[124] Duriau V.J., Reger R.K., Pfarrer M.D. (2007). A Content Analysis of the Content Analysis Literature in Organization Studies: Research Themes, Data Sources, and Methodological Refinements. Organ. Res. Methods.

[125] Longsheng X., Hui Z. (2022). Senior Executive Dual Environmental Cognition, Green Innovation and Enterprise Sustainable Development Performance. Econ. Manag..

